# Integration of homogeneous structural region identification and rock mass quality classification

**DOI:** 10.1098/rsos.181353

**Published:** 2019-01-09

**Authors:** Qingfa Chen, Tingchang Yin

**Affiliations:** 1 College of Resources, Environment and Materials, Guangxi University, Nanning, Guangxi 530004, People's Republic of China; 2 State Key Laboratory of Geomechanics and Geotechnical Engineering, Institute of Rock and Soil Mechanics, Chinese Academy of Sciences, Wuhan 430071, People's Republic of China

**Keywords:** three-dimensional joint network model, homogeneous structural region, modified blockiness index, rock mass rating, *RMR_MBI_*

## Abstract

In rock engineering projects, professionals assess the overall rock mass qualities using a sole value. However, the true qualities of partial rock masses are incompatible with such a value. To address this problem, the idea of regionally classifying rock mass qualities is proposed and the associated procedure presented. To achieve this goal, the probabilistic and deterministic joints within the study area were determined, and a three-dimensional joint network model was created. Then, the three-dimensional joint network model was discretized into interlocking subdomains, and the modified blockiness index (*MB_i_*) was used to finely identify the homogeneous structural regions, together with the *k*-means algorithm and the sum of squared errors (SSE). A synthetic model comprising homogeneous structural regions was developed and validated with respect to the extracted cross-sections. Next, an improved rock mass rating system (*RMR_mbi_*) was introduced, and the viability of *RMR_mbi_* was supported through a significant amount of theoretical cases and several real cases. Finally, visualization of regional *RMR_mbi_* classification results was performed. Results show that: (i) the homogeneous structural regions are finely demarcated in three dimensions, and (ii) the proposed idea can overcome the problem of rock mass quality classification using the conventional approach often leading to ‘overgeneralization’.

## 1. Introduction

Rock mass quality classification is an auxiliary tool frequently used as an indicator for rock engineering, in which various rock materials and/or rock mass properties are quantified and subsequently combined using addition and subtraction [1], multiplication and division [2], etc., and therefore, the overall quality of a given rock mass can be numerically assessed. A major benefit of such a classification system is that it can provide empirical guidance for designing rock engineering structures (e.g. excavation spans) [3] and also enable the estimation of rock mass mechanical properties, e.g. rock mass strength and rock mass deformation modulus [4].

Typically, rock mass quality classification results cannot be elaborated to a metre range [5], e.g. a rock mass rating (*RMR*) value always summarizes the average quality of the rock masses in a segment of the surrounding tunnel. For rock engineering projects (e.g. a rock dam or underground opening), engineers often assess the overall rock mass quality using a sole value, which, as in this case, may be problematic as the true qualities of the partial rock masses are incompatible with such a value. Some investigations have been undertaken to address this problem, e.g. Shang *et al*. [6] regionalized rock masses through engineering geological rock groupings in conjunction with lithologies and then conducted rock mass quality ratings in different regions. However, lithological regionalization does not consider rock mass structure, and even if the lithologies are identical or similar, rock mass qualities vary with different degrees of rock mass jointing. Objectively, a guideline should be followed when rock mass quality classification is conducted, i.e. regionalizing rock masses and then classifying rock mass qualities. Because lithological regionalization is slightly insufficient, the authors believe that the homogeneous structural region [7] (a further geological zoning) may be more appropriate, i.e. identifying rock mass homogeneous structural regions then classifying rock mass qualities.

Many existing identification methods of homogeneous structural regions zone engineer rock mass from a two-dimensional perspective, i.e. a rock outcrop, and use the joint data measured in daylighting rock masses, including joint orientation and joint density, to divide a given rock mass into different domains together with statistical knowledge. These existing methods mostly focus on small-scale joints (a trace length of approx. 4 m or shorter) [8,9], which are widely used in hydraulics, particularly the creation of a discrete fracture network (DFN) model and the subsequent seepage analysis. However, in these existing methods, some large-scale joints (e.g. incipient discontinuities, minor faults and bedding planes) are always ignored or defined as the presumptive boundaries of a homogeneous structural region [10,11]. Therefore, the existing methods cannot consider the implications of large-scale joints.

Additionally, some traditional rock mass quality classification systems, such as *RMR* [1], are limited in terms of quantification of the degree of jointing, i.e. the incorporation of rock quality designation (*RQD*) [12], which is frequently used in the rock engineering field but has some drawbacks as follows [13,14]: (i) *RQD* only counts the core pieces longer than 100 mm and fails to consider the effect of block size [15], and (ii) the *RQD* value is sensitive to orientation (i.e. orientation bias). Lowson & Bieniawski [4] recommended replacing the combined use of *RQD* and joint spacing with a sole use of joint frequency in the *RMR* system and stated that the limitations associated with the use of *RQD* can be removed. This stance is very controversial [16], and joint frequency is also orientation-dependent.

In this study, to overcome the aforementioned problems, a modified blockiness index (*MB_i_*) [17] was introduced, and attempts were made to finely demarcate the homogeneous structural regions in the study area (i.e. an underground mining stope). Subsequently, an improved *RMR* system (*RMR_mbi_*), in which the combined use of *RQD* and joint spacing was replaced with *MB_i_*, was adopted to three-dimensionally assess rock mass qualities. This study provides robust guidance for underground mine support and numerical simulation of rock mass behaviour (e.g. the discrete element model).

## 2. Material and methods

**Figure 1.**
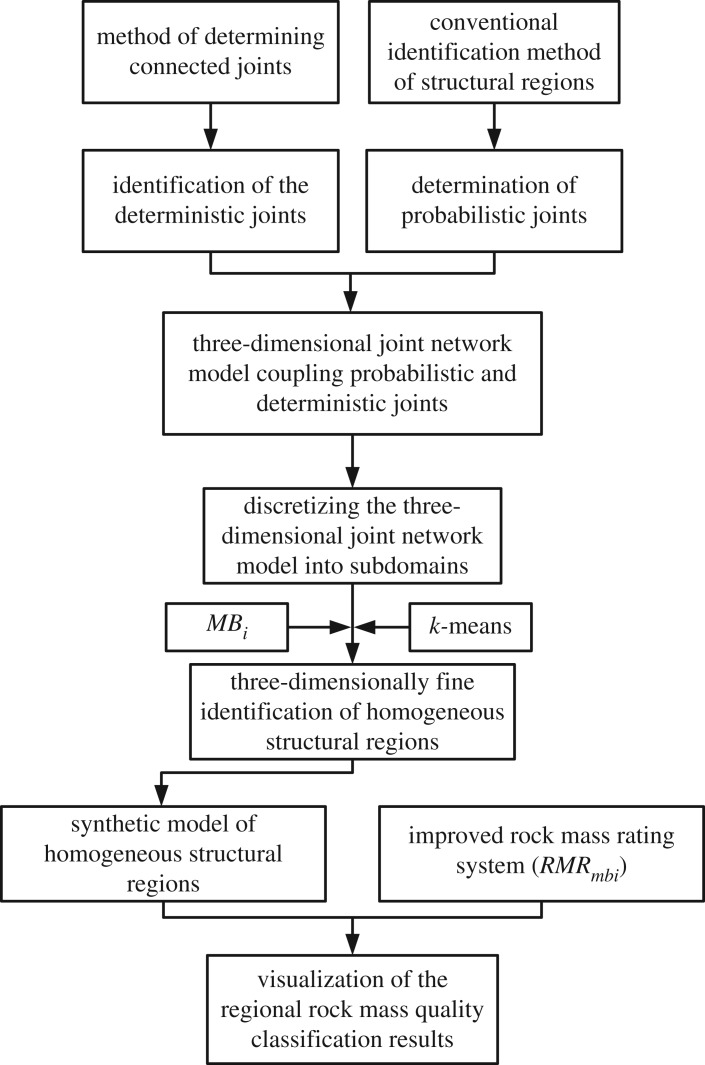
Procedure for regionally classifying rock mass qualities.

As described in §1, professionals always evaluate the overall rock mass qualities using a sole value, and this approach is often one of ‘overgeneralization’. To address this problem, an idea of regionally classifying rock mass qualities, i.e. identifying rock mass homogeneous structural regions then classifying rock mass qualities, is proposed. Based on this idea, the associated procedure was established and can be outlined as follows: (i) using an existing identification method of homogeneous structural regions and considering the implication of large-scale joints, the given rock mass was finely regionalized using the *MB_i_* index, and (ii) a visualization of overall rock mass qualities differentiated by *RMR_mbi_* values was performed. The flowchart for regionally classifying rock mass qualities is shown in figure 1, and details can be found in §§3–6.

## 3. Study area

**Figure 2.**
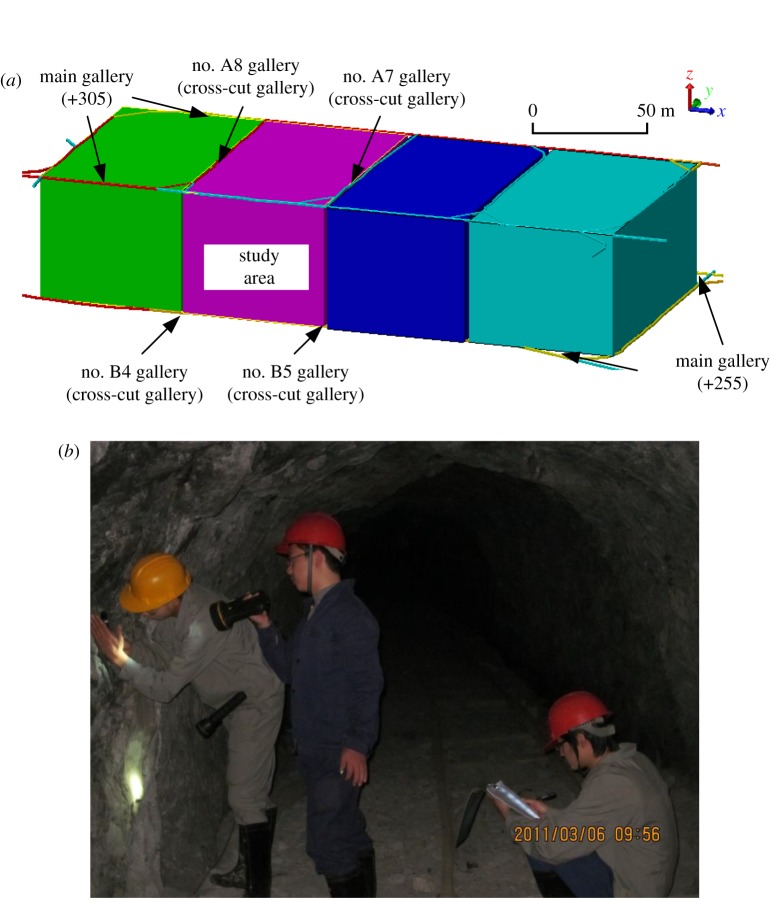
(*a*) Location of the study area; and (*b*) field observation.

The Zinc-polymetallic Mine is in Tongkeng, Guangxi Province, in Southwest China, and is rich in tin, lead, zinc, antimony and indium. It is also a large production base of non-ferrous metals in China. Faults are well developed in the Zinc-polymetallic Mine, and the ore bodies mainly occur in marlstone, calcareous marl, shale and so on. An underground mining stope (with a size of approximately 60 × 100 × 50 m) was selected as the testing area (figure 2*a*), and the field observation was carried out (figure 2*b*).

### 3.1. Determination of the distribution parameters of probabilistic joints

**Figure 3.**
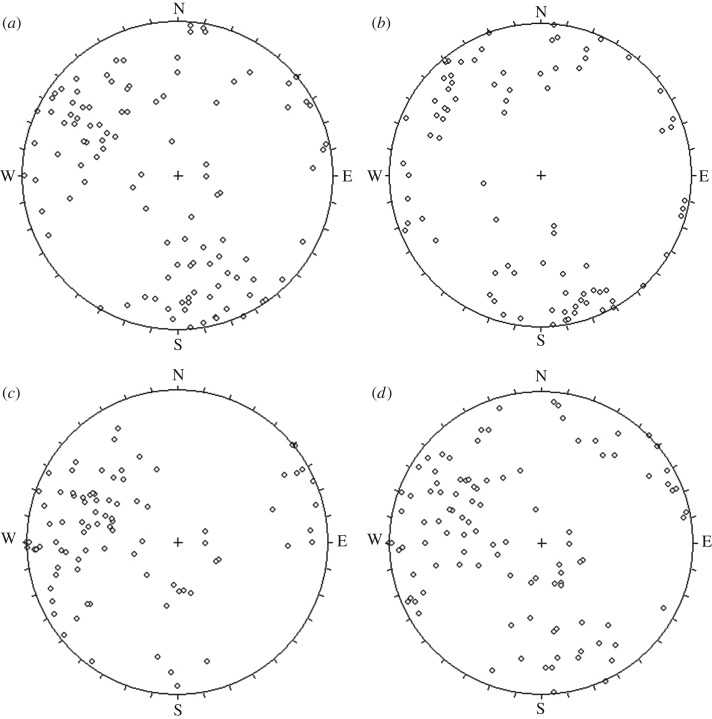
Schmidt lower hemisphere equal-area projections of the joint orientation data: (*a*) No. A7 gallery, (*b*) No. A8 gallery, (*c*) No. B4 gallery, (*d*) No. B5 gallery.

The existing identification method of homogeneous structural regions mostly focuses on examining the homogeneous degree of joints between two sites, and the tested joints are always small-scale (i.e. mechanical joints with a trace length of approximately 4 m or shorter) and intersect inside the rock mass in a significant amount. The geometrical parameters of small-scale joints can always be said to have a probabilistic distribution, and hence, when a joint network model is created, a large number of small-scale joints are randomly generated via a distribution function, which corresponds to the *in situ* conditions examined, and are termed ‘probabilistic joints’ [18]. Regarding that, the small-scale joints in the four cross-cut galleries were measured, and the orientation data are shown in figure 3.

Sufficient samples were obtained in the criss-crossed galleries, which enabled a test to determine the homogeneous degree of all the observed small-scale joints. The method proposed by Li *et al*. [7] was employed to test such a degree. It can be concluded that all the statistical *p*-values calculated are greater than 0.05, and hence no significant difference exists between these samples, which indicates that the small-scale joints in the test area are statistically homogeneous.

**Figure 4.**
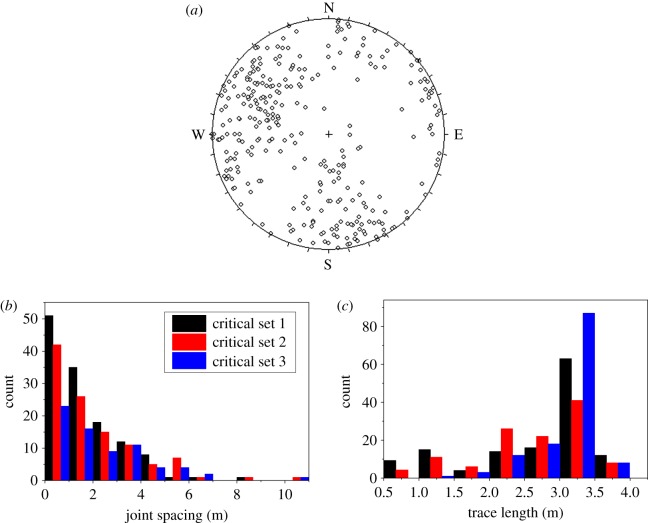
Overall small-scale joint data of the study area: (*a*) Schmidt lower hemisphere equal-area projections of the joint orientation data; (*b*,*c*) histograms of the joint spacing and trace length, respectively.

Overall small-scale joint data are presented in figure 4, and the probabilistic distribution parameters of these joints were determined as shown in table 1, which can be used to generate probabilistic joints.

**Table 1.** Some distributed parameters of the small-scale joints in the study area.

joint set no.	1	2	3
mean dip direction (°)	124.2977	0.1347	69.7002
mean dip angle (°)	56.4088	49.1857	88.6918
distribution type of trace length	normal	normal	normal
distribution type of joint orientation	Fisher	Fisher	Fisher
Fisher value (*κ*)	9.5019	5.4243	10.4184
three-dimensional density (m^3^)	0.0203	0.0150	0.0129

### 3.2. Identification of the deterministic joints and their geometrical properties

Some joints have been observed that are greater than the gallery scale. Investigation shows that these joints mostly belong to incipient discontinuities, minor faults, bedding planes, etc. Because the quantity is relatively small, these large-scale joints can be defined as ‘deterministic joints’ [19].

It is possible to define the diameter of a large-scale joint as infinite (note that the disc-joint model is used for creating a joint network). However, if all the observed joints that are greater than the gallery scale are generated as an infinite plane, the computer calculation is very time-consuming, and the intensity of jointing will be overestimated because the majority of large-scale joints may be connected. Therefore, a determination method for connected joints was employed, which enables the identification of the connected large-scale joints in an underground mining stope. In this method, the connectivity degree of large-scale joints can be examined with respect to joint categorization, mechanical property, coplanar condition, etc. (more details are provided in the electronic supplementary material).

**Figure 5.**
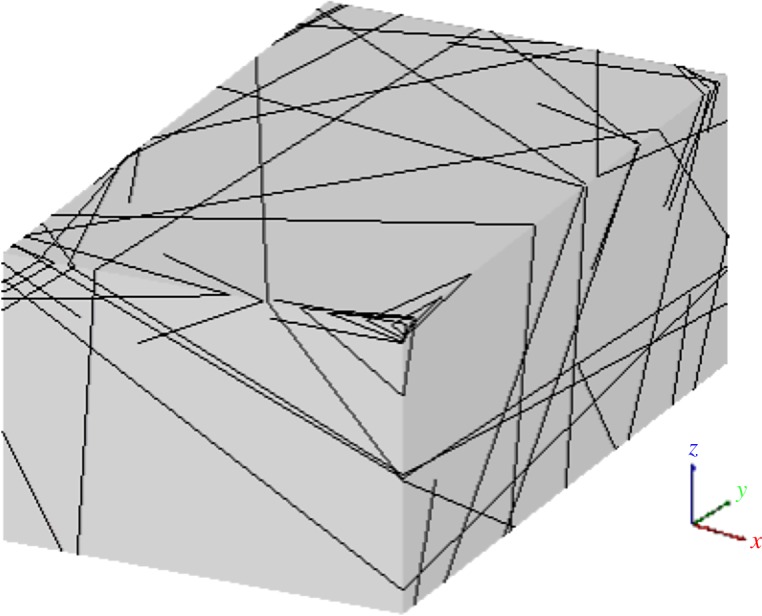
Three-dimensional joint network generated via deterministic joints.

Following the determination method of connected joints, the connectivity degrees of large-scale joints were investigated, and a total of 42 deterministic joints were identified. Most of them are incipient discontinuities, minor faults and bedding planes, with lithologies of limestone and silicite. The fillings are mainly calcite, and the joint wall is smooth or rough. The groundwater conditions are completely dry. The deterministic joints can be generated using five input parameters: *x*, *y*, *z*, *α*, and *β*. (*x*, *y*, *z*) denotes the coordinate of the disc-model centre, *α* is the dip direction, and *β* is the dip. The deterministic joint network is shown in figure 5.

### 3.3. Three-dimensional joint network model coupling probabilistic and deterministic joints

**Figure 6.**
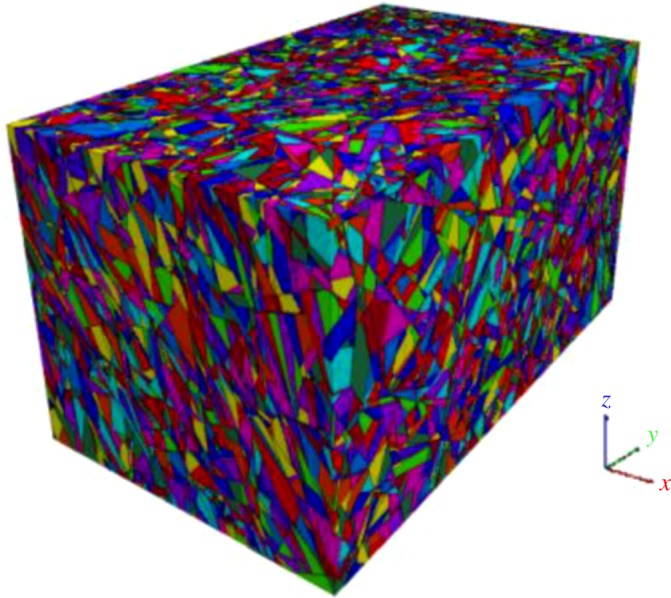
Three-dimensional joint network generated by the coupling of probabilistic and deterministic joints.

Typically, when the small-scale joints show a statistically homogeneous feature, the tested samples (galleries) can be treated as identical homogeneous structural regions. However, the large-scale joints should not be ignored, and thus, a further identification of homogeneous structural regions should be conducted. Before proceeding with this further identification, a three-dimensional joint network model coupling probabilistic and deterministic joints (with a size of 60 × 100 × 50 m) was developed (figure 6). Note that the generated range of probabilistic joints was 80 × 120 × 70 m, because there are some joints whose disc-model centres are outside the model but intersect the model, i.e. boundary effects were eliminated.

## 4. Three-dimensionally fine identification of homogeneous structural regions

### 4.1. Selection of the identification index and rationality

Generally, various geometrical parameters, including joint orientation, trace length and joint density, can be used to identify structural region boundaries. In this study, the given rock masses were finely regionalized in three dimensions using the *MB_i_* index. The *MB_i_* index is defined as the ratio of the volume of rock blocks, which are fully enclosed by joints, to the total rock mass volume, and it can be calculated as follows:

4.1 $$MB_{i}=B_{1}+\frac{1}{2}B_{2}+\frac{1}{3}B_{3}+\frac{1}{4}B_{4}+\frac{1}{5}B_{5},$$

where *B*_1_, *B*_2_, *B*_3_, *B*_4_ and *B*_5_ are the block percentages of the volumes in five intervals (0–0.008, 0.008–0.03, 0.03–0.2, 0.2–1.0 and greater than 1.0 m^3^), respectively. A higher *MB_i_* value indicates a more fractured rock mass and vice versa.

Over the years, joint orientation has typically been used to evaluate homogeneous structural regions, though it is not a prerequisite [20]. For example, Kulatilake *et al*. [21] used the box fractal dimension as a measure of a statistically homogeneous rock mass; they also reported that block sizes are primarily controlled by joint persistence and density.

The *MB_i_* index is a three-dimensional measurement of block size and degree of jointing that can capture the effects of joint persistence and density [22,23]. Meanwhile, the *MB_i_* value is produced by the joint network, which can be exactly adopted for structural region identification based on the three-dimensional joint network model coupling the probabilistic and deterministic joints.

### 4.2. Discretizing the three-dimensional joint network model into subdomains

**Figure 7.**
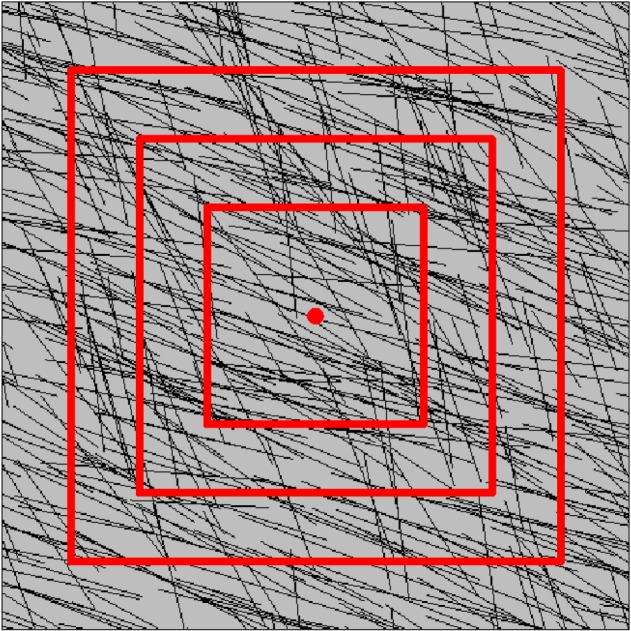
A two-dimensional hypothetical example to illustrate the children models. Children models of increasing sizes were developed along the geometrical centre of the parent model.

**Figure 8.**
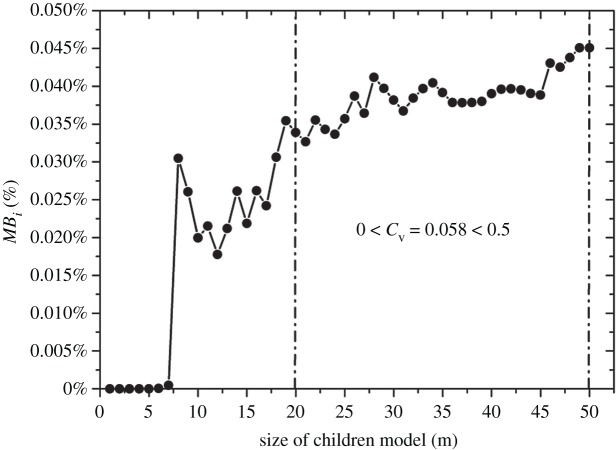
Determination of the optimal size of the children model (i.e. subdomain).

Before discretizing the three-dimensional joint network model (figure 6) into subdomains, the size of the subdomain needs to be determined. Referring to the concept of representative elementary volume (REV) [23], the optimal size of the subdomain was derived via the following steps: (i) the children models of the gradually increasing sizes were generated inside the parent model, as shown in figure 7, (ii) the *MB_i_* values of all the children models were calculated using equation (4.1), and (iii) the optimal size of 20 m was determined using the coefficient of variation (*C*_v_), as shown in figure 8. It is noted that the deterministic joints are temporarily excluded in the parent model, because if the deterministic joints are considered, the whole joint network model is no longer statistically homogeneous and the determined REV size is meaningless [18].

**Figure 9.**
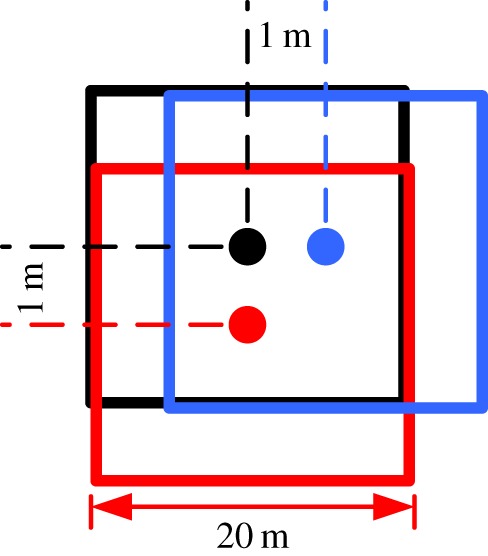
A two-dimensional hypothetical example to illustrate the interlocking (partially overlapped) subdomains. The three subdomains are highlighted with three colours, the distance between the two subdomains is 1 m, and the side length of a subdomain is 20 m.

**Figure 10.**
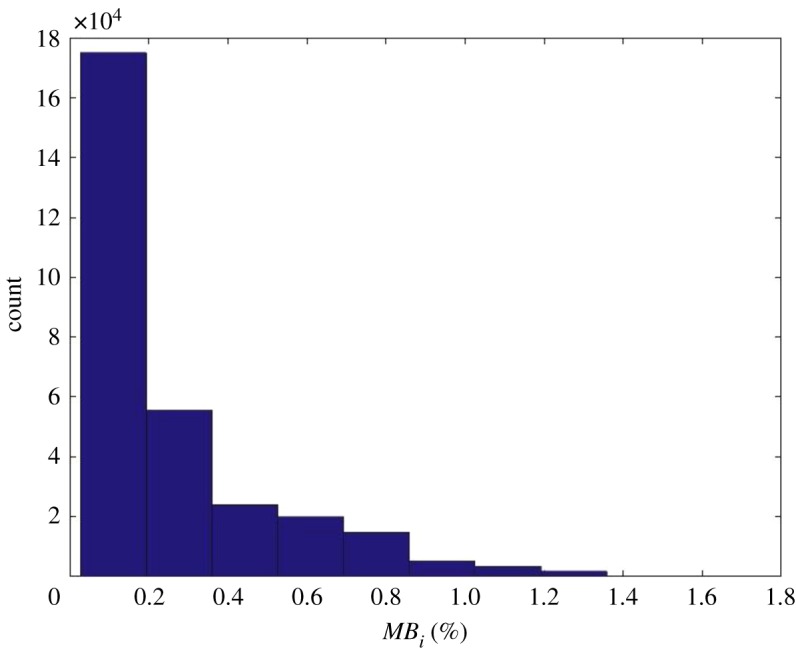
Histogram of the *MB_i_* values of all subdomains.

The interlocking subdomains that are partially overlapping each other were generated within the three-dimensional joint network model, and the distance between the two adjacent subdomains is 1 m (figure 9). Additionally, to measure the *MB_i_* values of the model boundaries, all the deterministic and probabilistic joints were generated and accommodated in a space of 80 × 120 × 70 m, and the true model size was 60 × 100 × 50 m. The *MB_i_* values of all subdomains were determined, as shown in figure 10, and have a normal distribution with parameter *μ* of 0.2703 and *σ*^2^ of 0.2721.

### 4.3. Finely identifying homogeneous structural regions using *k*-means and sum of squared errors

Cluster analysis is a process that partitions objects into *a* groups, and the yielded groups are generally called clusters. Objects in the same cluster are more similar, in some sense, to each other than to those in the other clusters. Cluster analysis is essentially similar to homogeneous structural region identification. To measure the similarity degree between different subdomains, a *k*-means algorithm [24] was used to cluster the *MB_i_* values of all the subdomains.

The *k*-means algorithm is a widely used clustering procedure. Before the *k*-means algorithm is executed, the cluster number should be determined. A classical *k*-means algorithm can be performed via the following steps: (i) randomly distribute the sample {*x*_1_, *x*_2_, … , *x_n_*} to *k* clusters and calculate the initial centre of each cluster {*z*_1_, *z*_2_, …, *z_k_*} using equation (4.2) as follows:

4.2 $$Z_{i}=\frac{1}{n}\sum_{x_{i}\in s_{i}}x_{i},$$

where *n* is the number of objects in cluster *i* and *s_i_* denotes cluster *i*; (ii) compute the distances between the object *x_i_* and *Z_i_*(*i* = 1, 2, … , *k*), and allocate this object to its nearest cluster; (iii) update the centre of the *k* cluster using equation (4.2); and (iv) calculate the *D* value using equation (4.3) as follows:

4.3 $$D=\sum_{i=1}^{n}\left[\mathrm{mi}n_{r=1,2,\ldots ,k}d{\left(x_{i},z_{i}\right)}^{2}\right],$$

and (v) end the algorithm if the *D* values converge, and if not, return to step (ii).

In this section, the sum of squared error (SSE) was adopted to determine an optimal cluster number, which can be calculated as follows:

4.4 $$\mathrm{SSE}=\sum_{i=1}^{k}\sum_{x\in s_{i}}^{}d{\left(x_{i},z_{i}\right)}^{2}.$$

**Figure 11.**
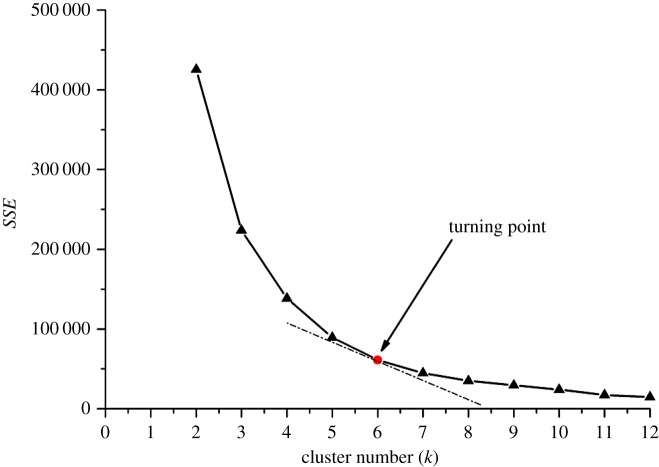
Optimal cluster number.

When the cluster number increases, the number of objects in a cluster will decrease, and the distances between the objects and the corresponding centres of the clusters will shorten. In this circumstance, SSE values will decrease. However, when the decrease degree of SSE values lessens, i.e. the slopes of the curve (SSE versus cluster number) insignificantly vary, a conclusion can be reached that increasing the cluster number can no longer improve the clustering qualities, and the corresponding data point can be deemed the turning point in the curve. Thus, the associated cluster number can be regarded as optimal. A scatter plot of SSE and cluster number was developed as shown in figure 11, indicating that the optimal cluster number is six. Therefore, a *k*-means algorithm with a cluster number of six was executed, and six clusters were yielded as follows: [0.02747%, 0.14986%], [0.14987%, 0.28447%], [0.28448%, 0.47531%], [0.47532%, 0.68421%], [0.68422%, 0.96486%] and [0.96487%, 1.91390%]. These concurrently suggest that the data values in the same cluster are highly similar, and the associated subdomains can be grouped into a homogeneous structural region.

### 4.4. Synthetic model of homogeneous structural regions and its verification

**Figure 12.**
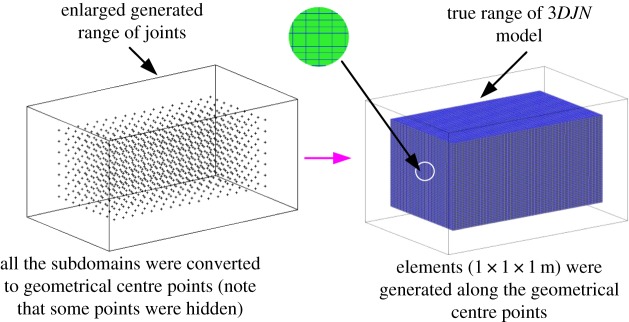
Pre-processing of subdomains.

**Figure 13.**
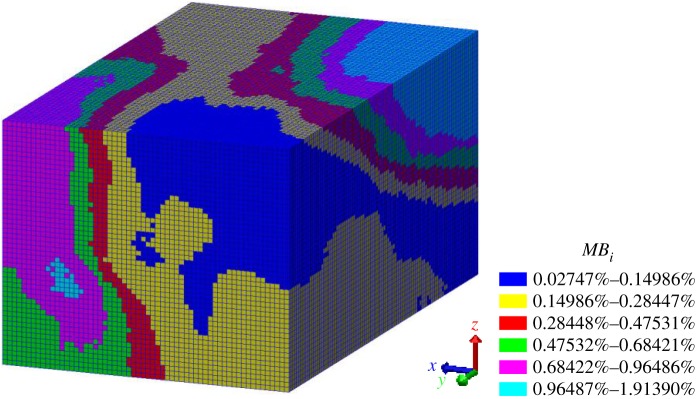
Synthetic model of homogeneous structural regions (60 × 100 × 50 m).

**Figure 14.**
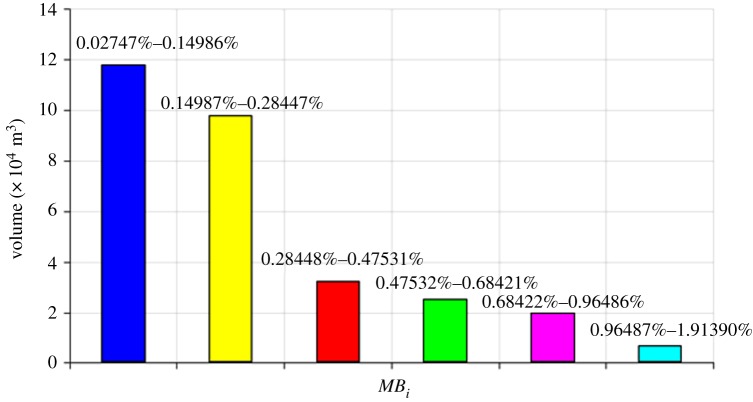
Histogram of the rock mass volumes in different homogeneous structural regions.

Pre-processing of subdomains was performed: all the subdomains were contracted into elements (or say, sub-subdomains) of 1 × 1 × 1 m along the centres of the subdomains, as shown in figure 12. In turn, the model (figure 6) was discretized again into elements. Subsequently, these elements were stained different colours according to their associated *MB_i_* values, i.e. *MB_i_* values in the same cluster were highlighted with an identical colour, as shown in figure 13. The histogram of the rock mass volumes in different homogeneous structural regions is shown in figure 14.

As can be seen in figures 13 and 14, the vast majority of rock masses in the study area fall into the intervals [0.002747%, 0.14986%] and [0.014987, 0.28447%]. If the joint network model of the study area is entirely built by probabilistic joints, the *MB_i_* values of the children models range from 0.033% to 0.045% (as shown in figure 8), which falls into the interval [0.014987, 0.28447%]. Thus, it can be concluded that the *MB_i_* values of the whole study area are majorly affected by probabilistic joints. However, it is improper to evaluate the overall study area as a homogeneous structural region, because the *MB_i_* values of the children models in the probabilistic joint network vary within a narrow interval. Figure 14 shows that a substantial proportion of subdomains have higher *MB_i_* values than the children models of the REV sizes, and this is a result of a large number of deterministic joints that enhance the degree of jointing. Nevertheless, the existing identification method of structural regions is limited in this respect.

**Figure 15.**
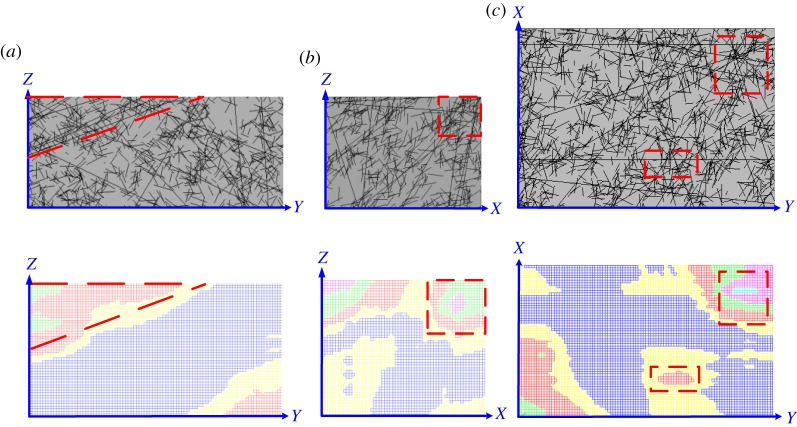
Graphical comparison test. (*a–c*) denote the cross-sections extracting along the three-dimensional joint network model (figure 6) and the synthetic model of homogeneous structural regions (figure 13) at half length, half width and half height.

Cross-sections were extracted along the three-dimensional joint network model (figure 6) and synthetic model of the homogeneous structural regions (figure 13) at half length, half width and half height, and comparisons were conducted to validate the accuracy of the fine identifications of homogeneous structural regions (figure 15). Visual inspections were performed, based on the dense degree of joints; as shown in the closed red (dashed) wireframes in figure 15, where the dense degree of joints is high, the *MB_i_* value is high. In short, this synthetic model of homogeneous structural regions can capture the differences in joint densities between subdomains, and its accuracy is supported.

## 5. A rock mass quality classification system: *RMR_mbi_*

The failure to accurately assess rock mass qualities may be a result of an unreliable measurement of block size [13]. Some drawbacks of the simultaneous use of *RQD* and joint spacing in the *RMR* system have been widely acknowledged as follows: (i) both *RQD* and joint spacing are anisotropic, (ii) the *RQD* concept ignores the block size effect [15], (iii) joint persistence is neglected, and (iv) the simultaneous use repeatedly calculates the joint density [25].

In this instance, the *RMR_mbi_* was introduced, in which the simultaneous use of *RQD* and joint spacing is replaced with *MB_i_* and the other input parameters remain unchanged. The *RMR_mbi_* has several advantages compared to the *RMR* [17,23] as follows: (i) *MB_i_* is a three-dimensional quantification of jointing degree and is not anisotropic, (ii) *MB_i_* counts blocks of all sizes, (iii) joint persistence is considered, and (iv) the *RMR_mbi_* does not repeatedly calculate the joint density. The rating of *MB_i_*(*R_M_*) can be determined using a continuous function as follows:

5.1 $$R_{M}=40-0.4\times MB_{i}.$$

The higher the *MB_i_* value, the higher the degree of jointing, and the poorer the rock mass quality.

### 5.1. Preliminarily analysing the viability of *RMR_mbi_* based on theoretical discrete fracture network (DFN) models

**Figure 16.**
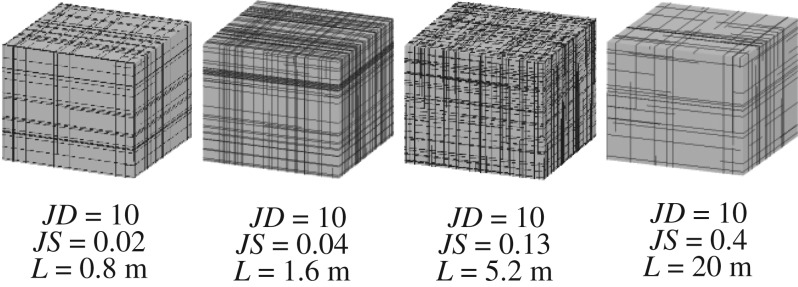
Some theoretical DFN Models (note that *JD* denotes the joint persistence, *JS* denotes the joint spacing, and *L* denotes the side length of the model).

As described in this section, a significant amount of theoretical DFN models were created to preliminarily analyse the viability of *RMR_mbi_*. Because the degree of jointing (or block size) is primarily influenced by joint spacing and persistence [23,26], 20 joint spacing values and 10 joint persistence values were chosen (table 2) and then cross-joined, and in this manner, 200 combinations of joint spacing and persistence were obtained. Additionally, the other input parameters of DFN models were fixed, as shown in table 3. Based on the 200 combinations and the fixed parameters, 200 theoretical DFN models were established (figure 16).

**Table 2.** Selected representative values of joint spacing and persistence.

joint spacing classification [23]	extremely wide	very wide	wide	moderate	close	very close	extremely close
selected value (m)	7	4, 5 and 6	0.9, 1.3, 1.7 and 2	0.3, 0.4, 0.5 and 0.6	0.09, 0.13, 0.17 and 0.2	0.03 and 0.04	0.01 and 0.02

joint persistence classification [23]	very high	high	medium	low	very low		
selected value (m)	30 and 40	15 and 20	5 and 10	2 and 3	0.5 and 1

**Table 3.** Distribution of the joint parameters of the theoretical DFN Model.

joint set no.	set 1	set 2	set 2
distribution type of joint persistence	uniform	uniform	uniform
distribution type of joint orientation	uniform	uniform	uniform
*κ* for Fisher	0	0	0
average dip direction/dip (°)	0/90	90/0	90/90

**Figure 17.**
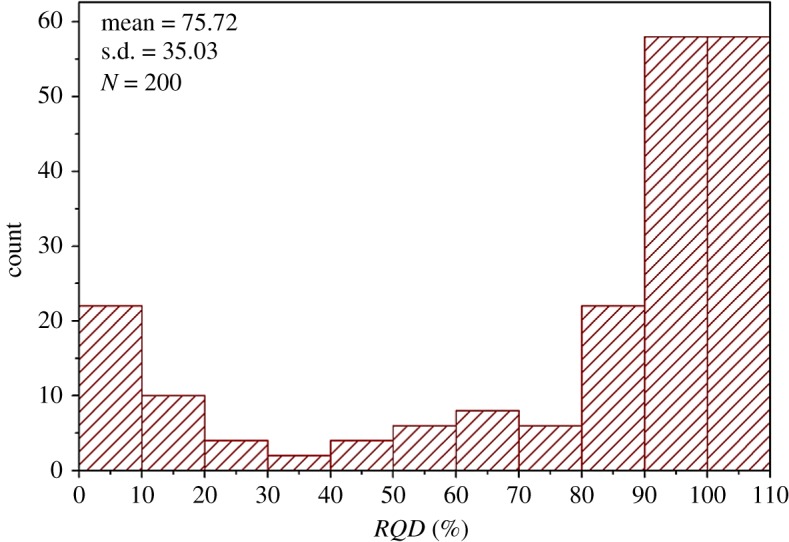
Histogram of the *RQD* values (theoretical DFN models).

**Figure 18.**
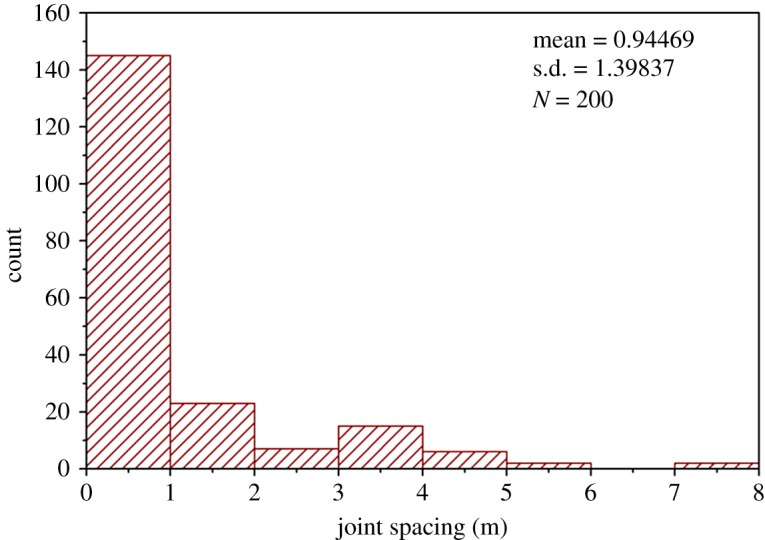
Histogram of the joint spacing values (theoretical DFN models).

**Figure 19.**
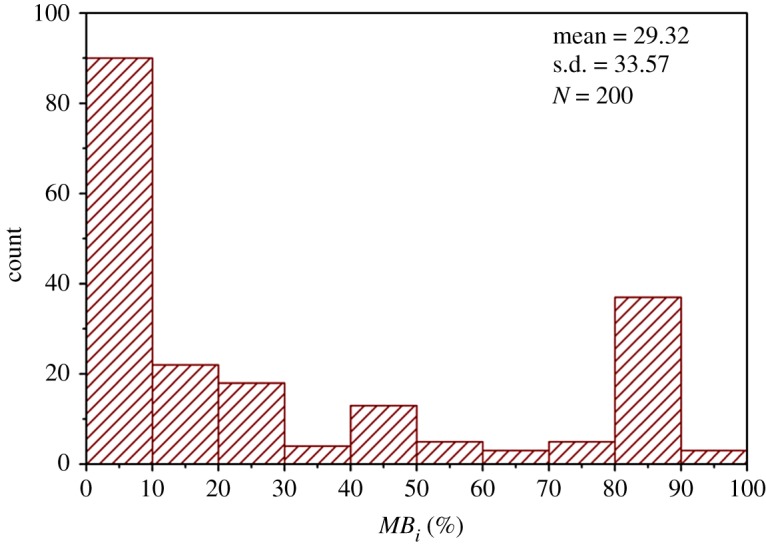
Histogram of *MB_i_* values (theoretical DFN models).

**Figure 20.**
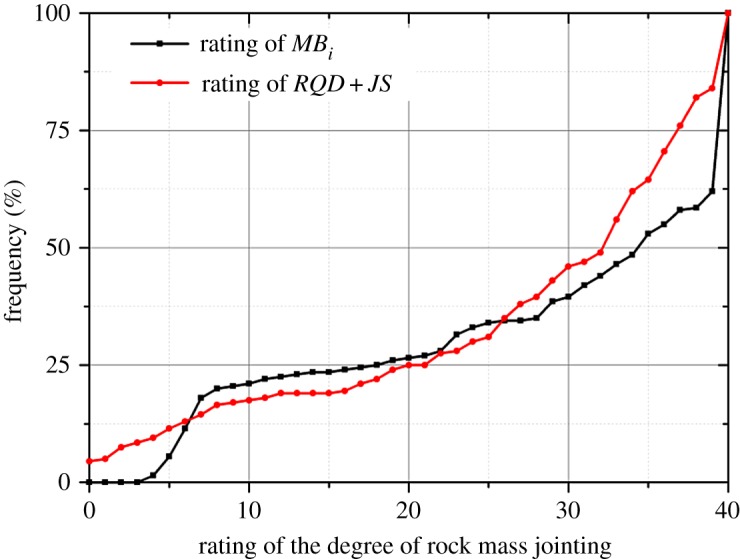
Comparison between *R_M_* and *R_RQD+JS_* (theoretical DFN models).

Because the *RMR_mbi_* and *RMR* only differ in the characterization of the degree of jointing, the *RQD* values and the joint spacing values of all theoretical DFN models were determined referring to [27], as shown in figures 17 and 18. Additionally, all the measured *MB_i_* values are shown in figure 19. Based on figures 17–19, the ratings of *RQD* plus joint spacing (*R_RQD+JS_*) and *MB_i_*(*R_M_*) were calculated, according to [28] and equation (5.1), respectively, as shown in figure 20. This figure shows that for the majority of theoretical DFN models, the *R_RQD+JS_* and *R_M_* share similar evaluation results, e.g. in the intervals of 10 to 30. However, for a number of theoretical DFN models, the two rating standards yield different results, which may be because of the implication of joint persistence on *MB_i_*. The correlation coefficient *r* [29] between *R_M_* and *R_RQD+JS_* was determined to be 0.93, which is very close to 1, and this suggests that (i) *R_M_* and *R_RQD+JS_* share a similar sensibility to distinguish rock mass structures, and (ii) the evaluation results of *R_M_* are in close proximity to those of *R_RQD+JS_*, which has been substantially applied, and therefore, *R_M_* is potentially adoptable.

### 5.2. Assessing the viability of *RMR_mbi_* based on several real data

**Figure 21.**
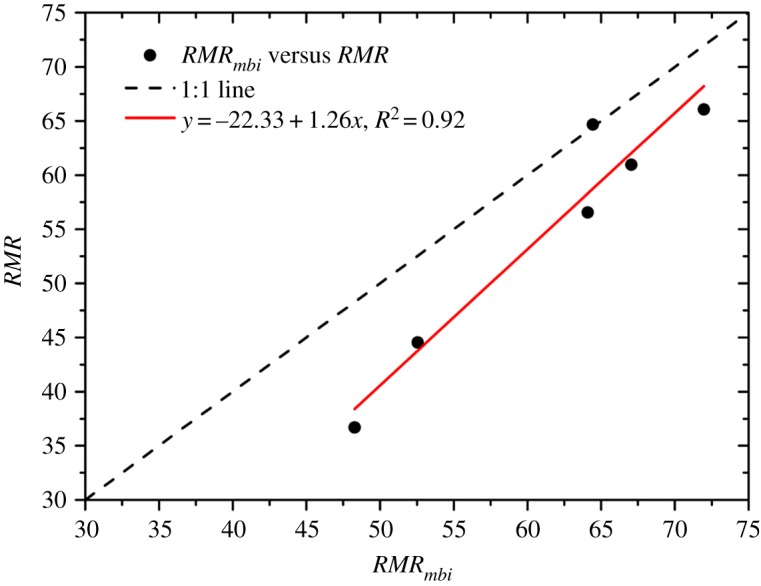
Comparison between *RMR_mbi_* and *RMR* (real data).

As described in this section, some real data (including all the input parameters of *RMR_mbi_* and *RMR*) collected from [30–33] were used to again support the viability of *RMR_mbi_*, and the final rating results are shown in figure 21. It can be seen that the fitting line is near the 1 : 1 line, suggesting that they have similar abilities to evaluate the qualities of real rock masses. However, the fitting line is under the 1 : 1 line, which indicates that most *RMR_mbi_* values are greater than those of *RMR*, and in turn, when appraising the rock mass qualities in the ‘Fair’ category or higher, the *RMR_mbi_* may not be as conservative, similar to *RMR*.

Overall, according to the results of the simulated experiments and real applications, it is considered that *RMR_mbi_* is a workable classification method with great application potential. *RMR_mbi_* not only overcomes the theoretical limitations of *RQD* and joint spacing but also produces practical and reasonable rating results. Figures 20 and 21 imply that *RMR_mbi_* is slightly different from *RMR*, and this circumstance is inevitable because the subsystem of *RMR_mbi_* (*R_M_*) can capture the influence of joint persistence on the degree of jointing.

## 6. Visualization of the regional rock mass quality classification results

**Figure 22.**
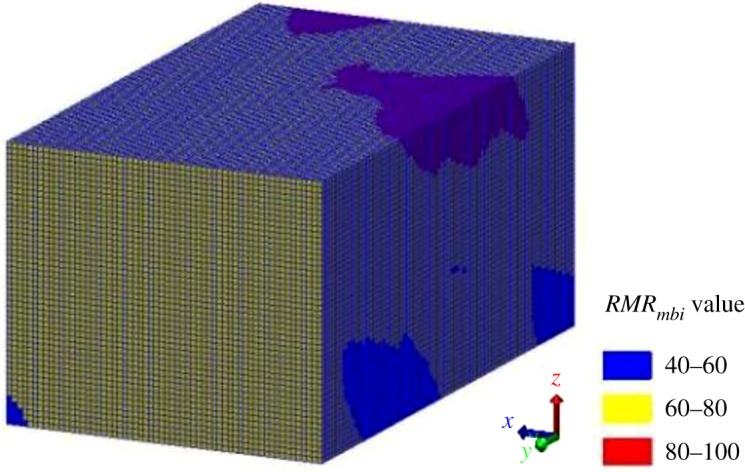
Visualization of the regional rock mass quality classification results of the study area.

As described in §4, the *MB_i_* values of all elements of the three-dimensional joint network model were obtained. Therefore, based on the synthetic model of homogeneous structural regions (figure 13), the *RMR_mbi_* classification and its visualization can be effectively implemented. During the geological investigation, a large number of stations were sited in the main and cross-cut galleries, and the geological data were measured. The Kriging method [11] was used to estimate the *RMR_mbi_* values of the untouchable rock masses, based on geological data measured in galleries. Then, the visual model of *RMR_mbi_* classification was constructed, which was stained with different colours according to the *RMR_mbi_* values (figure 22).

**Figure 23.**
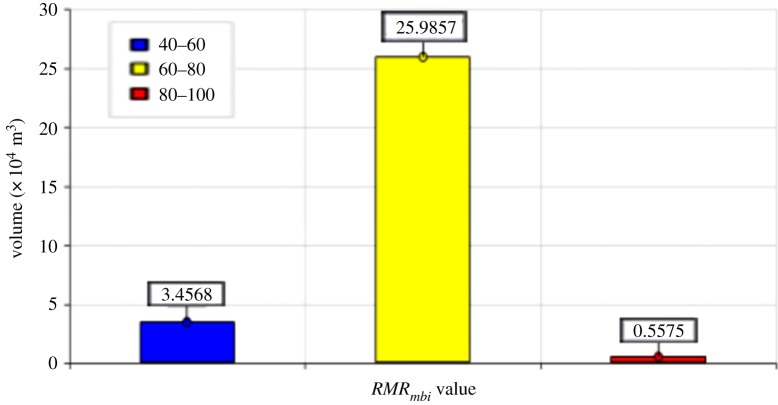
Histogram of volumes of rock masses in different *RMR_mbi_* classes.

As shown in figure 22, the rock masses of the study area are in Classes Ι, ΙI and III, respectively. The volumes of the rock masses of the different classes were counted, as shown in figure 23. The great majority of rock masses are in Class II followed by Classes III and Ι as follows: 25.9857 × 10^4^ m^3^ (Class II), 3.4568 × 10^4^ m^3^ (Class III) and 0.5575 × 10^4^ m^3^ (Class Ι). The rock masses in the three classes differ greatly in volume, indicating that Class II is the dominant quality of the study area. Additionally, the rock mass qualities gradually decrease with an increase in depth.

## 7. Conclusion

To address the problem that rock mass quality classification is often one of ‘overgeneralization’ when a traditional evaluation approach is used, the idea of regionally classifying rock mass qualities, i.e. identifying rock mass homogeneous structural regions then classifying rock mass qualities, was proposed and the associated procedure presented. Visualizations of homogeneous structural region identification were performed and rock mass classification results obtained. This work allows for several conclusions as follows:

(i) An existing evaluation method of structural regions was employed to determine the distribution parameters of probabilistic joints, and the deterministic joints were identified through a determination method of connected joints. Subsequently, a three-dimensional joint network model coupling probabilistic and deterministic joints was established.
(ii) The blockiness level of rock masses are primarily controlled by joint spacing and persistence; thus, these two joint properties can be considered in three dimensions if the *MB_i_* index is used to finely identify homogeneous structural regions.
(iii) The *k*-means and SSE were used together to cluster the measured *MB_i_* values of all subdomains, and optimal cluster numbers and clustering results were obtained. The *MB_i_* values in the same cluster can be said to have originated from an identical source (i.e. homogeneous structural region).
(iv) The three-dimensionally fine identification of homogeneous structural regions performed in this study can consider the implications of joint persistence and spacing, which have a strong identification ability. The identification results (synthetic model of homogeneous structural regions) can reflect the discontinuous and inhomogeneous features of natural rock masses.
(v) The consolidated use of *RQD* and joint spacing in the conventional *RMR* system was replaced with the *MB_i_* index, and this version was termed *RMR_mbi_*. Based on a large volume of theoretical DFN models and several real data, it is proved that *R_M_* and *R_RQD+JF_* share a similar sensibility to differentiate rock mass structures, and *RMR_mbi_* has a similar applicability compared to the *RMR* system. In other words, for the majority of rock masses, *RMR_mbi_* and *RMR* yield similar results, indicating that *RMR_mbi_* is adoptable; but owing to the difference in rock mass structure characterization, it is apparent that a slight discrepancy exists between the two systems. However, undoubtedly, the theoretical limitations caused by the combined use of *RQD* and joint spacing were tackled in the *RMR_mbi_* system that, as a consequence, have a strong application potential.
(vi) Based on the identification results of homogeneous structural regions, the *RMR_mbi_* system was applied to the study area, and a visual model of classification results of rock mass qualities was established. In this way, the complicated data (i.e. rock mass quality classification results) were converted to a three-dimensional digital model, which are beneficial in representing the spatial distribution of rock mass qualities and determining the engineering support schemes.
(vii) Based on the homogeneous structural region identification and rock mass quality classification system, we proposed a new method of regionally classifying rock mass qualities. However, it is still a generalization but with improvements, because it is not a true representation of the rock mass.

Additionally, the development of *RMR_mbi_* is a new attempt. Although the conventional *RMR* system has some limitations, it has been successfully applied for more than 40 years. The experience of its application has been substantially accumulated, which is exactly what the *RMR_mbi_* of the current version lacks. Furthermore, completing a conventional *RMR* task requires a few minutes or an hour in a local area; however, to obtain a final *RMR_mbi_* value, professionals may spend more time constructing joint network models and calculating rock block sizes (maybe several hours or days). Therefore, future studies are needed to further verify the applicability of *RMR_mbi_* (e.g. applying the *RMR_mbi_* to more real cases and developing a method to rapidly determine the *MB_i_* value of jointed rock masses).

## Supplementary Material

A method for determining connected joints

## Data accessibility

We include all the joint data in Dryad Digital Repository: http://dx.doi.org/10.5061/dryad.7nh05k6 [34].
