# Supplementary material for: Integration of homogeneous structural region identification and rock mass quality classification
Source: R Soc Open Sci. 2019 Jan 9;6(1):181353. doi: 10.1098/rsos.181353 (PMC6366223; doi:10.1098/rsos.181353)
Supplement: A method for determining connected joints [file rsos181353supp1.doc]

**Supplementary materials**

In this section, we present a method for determining connected joints. Using this method, the coplanar condition of joints can be analysed, the similarity degree of geological features of joints (λ) can be quantified, and the degree of connectivity of joints ($\delta$) can be determined.

**A1. Principle of determining connected joints**

**A1.1. Geometrical coplanar condition of two joints**

Joints were supposed to be infinite, and therefore, spatial relationships between two joints were assumed to be 1) parallel; 2) overlapped; and 3) intersected. If two joints overlapped, they were coplanar. Figure A1 provides an example demonstrating how to identify coplanar joints, which were formed as follows:

1. Suppose that *l* is the intersect line of two joints, i.e. f_1_ and f_2_, and A and B are points in the two joints, respectively.
2. Two segments (AC_1_ and BC_2_) were constructed perpendicular to *l* (C_1_ and C_2_ are the corresponding feet of the perpendiculars in *l*).
3. Two segments (A_1_C_2_ and B_1_C_1_) parallel to AC_1_ and BC_2_, respectively, were constructed and the length of A_1_C_2_/ B_1_C_1_ was equal to AC_1_/BC_2_.
4. Two segments (C_1_E and C_2_F) were constructed perpendicular to AB_1_ and A_1_B. E and F are the corresponding feet of the perpendiculars in AB_1_ and A_1_B, respectively. D was the point of intersection of the segments EF and AB, and D_1_ and D_2_ were the projections from point D onto the two joint planes (i.e. f_1_ and f_2_), respectively.
5. Segment D_1_E_1_ was constructed perpendicular to AC_1_. E_1_ was the foot of the perpendicular in AC_1_.

As shown in Figure A1, $\gamma_{1}$ and $\gamma_{2}$ are the included angles between joint planes f_1_ and f_2_ and segment AB, respectively; $\gamma_{1}^{'}$ and $\gamma_{2}^{'}$ are the included angles between joint planes f_1_ and f_2_ and segment AB_1_(or plane AB_1_BA_1_), respectively; $\theta$ is the included angle between f_1_ and f_2_; $\overrightarrow{\mathrm{n}_{1}}$ and $\overrightarrow{\mathrm{n}_{2}}$ are the normal vectors to joint planes f_1_ and f_2_, respectively; $\overrightarrow{\mathrm{c}}$ is a vector parallel to *l*, and $\overrightarrow{\mathrm{c}}=\overrightarrow{\mathrm{n}_{1}}\times\overrightarrow{\mathrm{n}_{2}}$; the Cartesian coordinates of A and B are (*x*_1_, *y*_1_, *z*_1_) and (*x*_2_, *y*_2_, *z*_2_), respectively; $\overrightarrow{\mathrm{a}}$ is the vector AB; and is the angle between $\overrightarrow{\mathrm{a}}$ and $\overrightarrow{\mathrm{c}}$ and is the co-angle.

Figure A1. Demonstration of the coplanar joints

$\overrightarrow{\mathrm{n}_{1}}$ and $\overrightarrow{\mathrm{n}_{2}}$ can be calculated as follows:

|  | $\overrightarrow{\mathrm{n}_{1}}\text{=}\left(\text{sinα}_{1}\text{sinβ}_{1}\text{,sinα}_{1}\text{cosβ}_{1}\text{,cosα}_{1}\right)$ | (A-1) |
| --- | --- | --- |
|  | $\overrightarrow{\mathrm{n}_{2}}\text{=}\left(\text{sinα}_{2}\text{sinβ}_{2}\text{,sinα}_{2}\text{cosβ}_{2}\text{,cosα}_{2}\right)$ | (A-2) |
|  | $\overrightarrow{\mathrm{a}}\text{ =}\left(\mathrm{x}_{2}-\mathrm{x}{}_{1},\mathrm{y}_{2}-\mathrm{y}_{1},\mathrm{z}_{2}-\mathrm{z}_{1}\right)$ | (A-3) |

where α_1_ and α_2_ are the dip angles of f_1_ and f_2_, respectively, and β_1_ and β_2_ are the dip direction of f_1_ and f_2_, respectively.

$\theta$, $\gamma_{1}$ and $\gamma_{2}$ can be determined as follows:

|  | $\theta\text{ = cos}^{\text{-1}}\left(\frac{\left\vert\overrightarrow{\mathrm{n}_{1}}\cdot\overrightarrow{\mathrm{n}_{2}}\right\vert}{\left\vert\overrightarrow{\mathrm{n}_{1}}\right\vert\cdot\left\vert\overrightarrow{\mathrm{n}_{2}}\right\vert}\right)$ | (A-4) |
| --- | --- | --- |
|  | $\gamma_{1}\text{= 90}^{^{\circ}}\text{ - cos}^{\text{-1}}\left(\frac{\left\vert\overrightarrow{\mathrm{n}_{1}}\cdot\overrightarrow{\mathrm{a}}\right\vert}{\left\vert\overrightarrow{\mathrm{n}_{1}}\right\vert\cdot\left\vert\overrightarrow{\mathrm{a}}\right\vert}\right)$ | (A-5) |
|  | $\gamma_{2}\text{= 90}^{^{\circ}}\text{ - cos}^{\text{-1}}\left(\frac{\left\vert\overrightarrow{\mathrm{n}_{2}}\cdot\overrightarrow{\mathrm{a}}\right\vert}{\left\vert\overrightarrow{\mathrm{n}_{2}}\right\vert\cdot\left\vert\overrightarrow{\mathrm{a}}\right\vert}\right)$ | (A-6) |

If the measurement errors are neglected, some conclusions can be drawn:

1. If $\gamma_{1}\text{=}0$, then $AB\in f_{1}$; if $\gamma_{2}\text{=}0$, then $AB\in f_{2}$; and if either $\gamma_{1}$ or $\gamma_{2}$ is equal to 0, then the other is certainly 0. If $\theta\text{=}0$ and $\gamma_{1}\text{=}0$ or $\gamma_{2}\text{=}0$ (i.e. $\theta\text{=}0$ and $AB\in f_{1}$ or $AB\in f_{2}$), then the two joints overlap.
2. If (*i*)$\gamma_{1}\neq0$ or , but $\theta\text{=}0$ and (*ii*) $AB\notin f_{1}$ or $AB\notin f_{2}$ (i.e. line AB intersects with the two joints), then the two joints are parallel.
3. If $\theta\neq0$, then the two joints intersect.

Therefore, the ideal condition of the two overlapped joints (i.e. two finite joints are coplanar) is as follows:

|  | $\left\{\begin{matrix} \theta=0 \\ \gamma_{1}=0\text{ }or\text{ }\gamma_{2}=0 \end{matrix}\right.$ | (A-7) |
| --- | --- | --- |

In practice, because of the measurement errors and complicated joint shape, numerous coplanar joints will be missed if Eq. (A-7) is used. Therefore, if the errors of $\theta$ and $\gamma_{1}$ or$\gamma_{2}$ are in a narrow range, the two joints can be said to be coplanar.

Eq. (A-4) shows that the error range of $\theta$ is determined by the measurement errors of dip and dip direction and the roughness degree of the joint wall. From Eqs. (A-5) and (A-6), the error ranges of $\gamma_{1}$ and $\gamma_{2}$ are determined by the measurement errors of the dip, dip direction, and the coordinates of A and B and the degree of roughness of the joint wall. The differences between the determinant factors of the two error ranges can be ignored, and the error ranges of $\theta$ and $\gamma_{1}$ or $\gamma_{2}$ are defined as identical values.

The improved coplanar condition is as follows:

|  | $\left\{\begin{matrix} 0\leq\theta\leq\mathrm{c} \\ 0\leq\gamma_{1}\leq\mathrm{c}\text{ or 0}\leq\gamma_{2}\leq\mathrm{c} \end{matrix}\right.$ | (A-8) |
| --- | --- | --- |

where *c* is a very small angle (described by °). The *c* value is determined by the degree of roughness of the joint wall profile, which can be quantified by the fluctuation angle of the joint wall profile *i*, as shown in Figure A2. *i* can be calculated as follows:

|  | $\mathrm{i}=\text{arctan}\left(\frac{2\mathrm{h}}{\mathrm{L}}\right)$ | (A-8) |
| --- | --- | --- |

where *h* is the mean relief height and *L* is the mean trace length. Therefore, the *c* value is equivalent to *i*.

Figure A2. Calculation of *i* value

**A1.2** **Similarity condition of the geological features of joints**

Occasionally, two joints satisfy Eq. (A-8) but are not connected. Figure A3 illustrates a case in which the two joints are coplanar but disconnected. The a is a hypothetical infinite plane that involves two finite joints f_1_ and f_2_, and A and B are the observed stations of f_1_ and f_2_, respectively. As shown in this figure, joints f_1_ and f_2_ are definitely coplanar but disconnected.

In a real engineering project, if the geological features of two coplanar joints are the same or similar, the two joints can be said to be connected or disconnected but within a very short distance. Owing to excavation activities and geotectonic movements, those disconnected coplanar joints with a very short distance may extend and then connect and be deemed connected joints. Therefore, one should test the degree of similarity of the geological features of the joints.

Figure A3. Two joints that are coplanar but disconnected

The main geological features of joints include genesis, mechanics, lithology, orientation, persistence, density, aperture, filling, morphology, intersected pattern, etc. Density and intersected pattern are not the features of a single joint, and orientation was considered in Section A1.1. In this study, the degree of similarity of the geological features of joints (λ) ranges from 0 to 1, and a lower λ value denotes a lower degree. A table analytical method is suggested, as shown in Table A1.

As shown in Table A1, the geneses of joint planes should be considered as a basic condition. Only if the basic condition is satisfied can a similarity test be continued, or the λ value is 0. In the third column of Table A1, all joint properties are assigned weights; in the sixth column, the rating score ranges from 0 to 1, and a lower score denotes a lower degree. Finally, the λ value can be determined via a sum of all the products of the scores and corresponding weights. If $\lambda\geq0.6$, then the two joints are similar with respect to geological features.

Table A1. Analytic table of the similarity degree of geological features of joints

| No. | Joint Property | Weight | Joint f_1_ | Joint f_2_ | Rating Score for Degree of Similarity | Score × Weight |
| --- | --- | --- | --- | --- | --- | --- |
| 1 | Genesis | *X*_1_ |  |  |  |  |
| 2 | Mechanics | *X*_2_ |  |  |  |  |
| 3 | Lithology | *X*_3_ |  |  |  |  |
| 4 | Persistence | *X*_4_ |  |  |  |  |
| 5 | Aperture | *X*_5_ |  |  |  |  |
| 6 | Morphology | *X*_6_ |  |  |  |  |
| 7 | Filling | *X*_7_ |  |  |  |  |
|  | Sum | 1 | / | / | / | λ |

**A2. Method for determining connected joints**

**A2.1. Determining condition**

According to the principle of determining connected joints, the determining condition of two connected joints is as follows:

|  | $\left\{\begin{matrix} 0\leq\theta\leq\mathrm{c} \\ 0\leq\gamma_{1}\leq\mathrm{c}\text{ or }0\leq\gamma_{2}\leq\mathrm{c}\text{ } \\ \lambda\geq0.6 \end{matrix}\right.$ | (A-9) |
| --- | --- | --- |

where *c* is a small angle.

**A2.2. Calculation of the degree of connectivity of two joints**

The degree of connectivity of joints that satisfies the determining condition, i.e. Eq. (A-9), can be quantified. From Figure A1, $\text{sin}\gamma_{1}=\frac{DD_{1}}{AD}$, $\text{sin}\gamma_{1}^{'}=\frac{EE_{1}}{AE}$, , and $DD_{1}=EE_{1}$, therefore

|  |  | (A-10) |
| --- | --- | --- |

In addition,

|  |  | (A-11) |
| --- | --- | --- |

Plane ABC_1_ was delineated, as shown in Figure A4. As shown in this figure, the projections of two joints are two straight lines, and the projection of the intersect line of two joints (i.e. *l*) is a point. When the included angle of two joints (i.e. f_1_ and f_2_) is$\theta$, the chord is segment AB, and an arc with a central angle of$2\theta$ can be constructed. Two reflectionally symmetrical arcs can be obtained, where the segment AB passes through and divides them. C_1_, C_2_, and C_3_ are the projections of *l* in these three cases, respectively. All the included angles are sharp.

If the projection of *l* is C_1_, then $\theta\text{ = }\gamma_{1}^{'}\text{+}\gamma_{2}^{'}$. From Eqs. (A-10) and (A-11), we see that

|  |  | (A-12) |
| --- | --- | --- |

If the projection of *l* is C_2_, then $\theta\text{ = }\left|\gamma_{1}^{'}-\gamma_{2}^{'}\right|$. From Eqs. (A-10) and (A-11), we see that

|  |  | (A-13) |
| --- | --- | --- |

If the projection of *l* is C_3_, then $\theta\text{ =180}^{\circ}-\text{ }\gamma_{1}^{'}-\gamma_{2}^{'}$. From Eqs. (A-10) and (A-11), we see that

|  |  | (A-14) |
| --- | --- | --- |

Four variables can be observed in Eqs. (A-12), (A-13), or (A-14), and three (i.e. $\gamma_{1}$, $\gamma_{2}$, and $\theta$) are independent. Therefore, the degree of connectivity of the two joints is calculated as follows:

|  | $\text{δ = }\sqrt{\left(1-\frac{\gamma_{1}}{\mathrm{c}}\right)\cdot\left(1-\frac{\gamma_{2}}{\mathrm{c}}\right)\cdot\left(1-\frac{\theta}{\mathrm{c}}\right)\cdot\lambda}$ | (A-15) |
| --- | --- | --- |

where $0\leq\gamma_{1}\text{ and }\gamma_{2}$, $\theta\leq\mathrm{c}$, $0.6\leq\lambda\leq1$, and $\delta$ is the degree of connectivity between the two joints. When , the connectivity of the two joints is optimal, and vice versa.
